# Fast-tracking development of homozygous transgenic cereal lines using a simple and highly flexible real-time PCR assay

**DOI:** 10.1186/1471-2229-13-71

**Published:** 2013-04-30

**Authors:** Jos C Mieog, Crispin A Howitt, Jean-Philippe Ral

**Affiliations:** 1 CSIRO Food Futures National Research Flagship, GPO Box 1600, Canberra ACT 2601, Australia

**Keywords:** Real-time PCR, qPCR, Cereal, Wheat, Transgene, Copy number, Homozygous line

## Abstract

**Background:**

A crucial step in the evaluation of newly produced transgenic plants is the selection of homozygous plants. Here we describe an efficient and highly flexible real-time PCR-based method for the development of homozygous lines in plant models with complex (multiple) genomes and/or relatively long generation times (>3 months) using direct copy number determinations.

**Results:**

An existing DNA extraction method was converted into a high-throughput plant leaf DNA extraction procedure yielding DNA suitable for real-time PCR analyses. Highly specific and efficient primer pairs were developed for a bread wheat reference gene (Epsilon Cyclase) and for standard sequence elements in the gene cassette routinely used for cereal transformations (an intron bridge and the Nopaline Synthase terminator). The real-time PCR assay reliably distinguished wheat plants with a single copy of the transgene from individuals with multiple copies or those lacking the transgene. To obtain homozygous lines carrying a unique insertion event as efficiently as possible, T_0_ plants (plants raised from transformed callus) with a single copy of the transgene were selected and their progeny screened for homozygous plants. Finally, the assay was adapted to work on rice.

**Conclusions:**

The ability to quickly, easily and accurately quantify the construct copy numbers, as provided by the real-time PCR assay, greatly improved the efficiency and reliability of the selection of homozygous transgenic plants in our case study. We were able to select homozygous plants in early generations, avoiding time-consuming methods such as large scale analysis of segregation patterns of descendants and/or Southern blotting. Additionally, the ability to specifically develop homozygous lines carrying a unique insertion event could be important in avoiding gene silencing due to co-suppression, and if needed assist in the selection of lines suitable for future deregulation. The same primer pairs can be used to quantify many different wheat transgenic events because the construct-specific primer pairs are targeted to standard sequence elements of the cereal gene cassettes, making the method widely applicable in wheat GM research. Moreover, because all procedures described here are standardized, the method may easily be adapted to vectors lacking the target regions used here and/or to other plant models.

## Background

Genetic modification, the insertion of a DNA construct into a host genome, is an emerging powerful tool for increasing productivity and/or product quality of crop plants [1]. However, the transformation process produces hemizygous plants (transgene is inserted without allelic counterpart) and the construct can be lost in subsequent generations through Mendelian segregation. Therefore, most downstream applications of genetic transformation require the transgene to be homozygous in the host genome. For models such as *Arabidopsis thaliana* with simple screening options such as marker selection [2], short generation times (<1 month), and single-to-low copy number transformation methods (through *Agrobacterium tumefaciens*), selection of homozygous plants can be achieved within respectable timelines through Mendelian segregation studies over few generations [3]. However, this approach is very slow and cumbersome for agricultural models such as wheat, barley, rice and corn.

Most wheat transformations to date have been achieved using particle bombardment [4] in which, in many cases, multiple copies of the construct are inserted, either randomly throughout genomes or within a single insertion site (linked copies). Multiple unlinked copies cause complex segregation patterns, potentially requiring screening over many generations to establish the zygosity of a line. Linked copies will segregate as a single copy and will therefore not be noticed in Mendelian segregation studies. However, multiple inserted copies (either linked or unlinked) have been implicated in issues with transgene silencing [5] and are not suitable for lines being considered for deregulation, indicating a need to identify linked copies. Recently, progress has been made on the efficiency of *A. tumefaciens* mediated transformation in cereals including wheat (reviewed by Sood *et al*. [4]), which may limit the number of copies inserted into the genome and therefore reduce (but not avoid) some of these complications. Nonetheless, the multi-copy issues together with the long generation times of most agricultural models (>3 months) highlight the need for a more efficient alternative to Mendelian segregation studies, such as direct copy number determination.

The two most popular methods of directly quantifying copy numbers of a DNA fragment (gene, construct, etc.) are Southern blot and real-time PCR (reviewed by Bubner and Baldwin [2]). Southern blotting is the original method and is commonly used in order to determine copy numbers of DNA fragments. It involves blotting of enzymatically digested genomic DNA followed by hybridisation of specific labelled DNA probe corresponding to the sequence of interest [6]. As powerful and reliable as the technique can be, this somewhat cumbersome technique displays some important limitations. Southern blot may fail to accurately assess the exact number of copies due to DNA alterations and/or loss of restriction site, or to distinguish copies that are situated closely together (in particular linked copies) [2]. In addition, Southern blots will not usually give information on zygosity, which is needed to select a homozygous plant in the T_1_ generation. Using this technique, information on zygosity would have to be gathered in the T_2_ generation by analyzing the segregation ratios of the trait of interest or additional Southern blots. Furthermore, Southern blotting requires each construct to have a specific and labelled probe, making it less suitable to screen sample collections containing multiple constructs.

Real-time PCR is based on the detection of fluorescence produced during the amplification process of the PCR. This fluorescence can be produced by an intercalating dye that fluoresces when bound to the double-stranded DNA (e.g. SYBR green), or by a probe containing both a fluorophore and a quencher (e.g. Taqman) that binds between the primers. During primer extension, the probe is broken down, releasing the fluorophore from the quencher. The result of a reaction is expressed in a C_T_ value which represents the Cycle number where the fluorescence overtakes a pre-set threshold value. The comparative C_T_ method is considered to be the most robust for copy number determination [2]. This technique requires the comparison of the C_T_ value from the gene of interest (GOI) to the C_T_ value of an endogenous reference gene. Benefits of this method are that no standard lines are needed and the DNA concentration is allowed to vary somewhat between samples, but it is crucial that both the target and reference reactions have near-identical amplification efficiencies. In theory, real-time PCR analyses will detect the inserted transgene regardless of where it is located within the genome.

Real-time PCR has been employed to determine transgene copy numbers in plants such as wheat [7,8], maize [9], rice [10], tomato [11] and sugarcane [12]. There are, however, concerns that real-time PCR is not accurate enough to reliably determine zygosity [7,13], especially when the number of technical replicates is constrained due to the large number of plants requiring screening. However, we believe that previous results that found real-time PCR unreliable for copy numbers analyses and/or zygosity determination were likely due to suboptimal optimization procedures that can be overcome. In this case, real-time PCR may have been under-utilized in respect to its capacity to assist in the establishment of transgenic homozygous lines, especially in plants with longer generation times.

In this paper, we describe a real-time PCR based method with the required accuracy and precision to be able to distinguish plants with only a single copy of a transgene from homozygous plants and/or plants with multiple insertions. We show how this information can be best used in the development of transgenic homozygous lines hosting a unique insertion event. The method is specifically designed to be highly versatile and all procedures are standardized and described in sufficient detail to facilitate its adaptation to new organisms and/or vectors.

## Methods

### Transgenic plants

In the first experiment, we used DNA samples from offspring of self-fertilized plants which originated from crosses between a Glucan, Water Dikinase (GWD) RNAi transformed, single-insertion homozygous wheat line (based on observed Mendelian segregation history over 5 generations by endpoint PCR, as described in Ral *et al.*[14]) and a wild type plant. The aim of this test was to determine if it was possible distinguish between single copy and homozygous plants using relative quantification (see Methods - real-time PCR copy numbers assay).

In a second experiment we followed four wheat plants, biolistically transformed with the RNAi cassette, over up to four generations (T_0_ to T_3_) to investigate the effectiveness of our assay in selecting homozygous transgenic lines.

### DNA isolation

DNA was isolated from leaf samples using a modified method obtained from Ellis *et al.*[15]. Ca. 1 cm of young leaf sample was put into a 96x1 mL masterblock (Greiner Bio-one) together with one 5 mm glass bead (Sigma-Aldrich). The block was sealed with a sealing mat (Thermo Scientific) and frozen for at least 1 hour at −80°C. Next, the frozen block was placed in a Retch MM300 ball mill and the tissue was pulverized for 1 min at maximum frequency (30/s). Three hundred μL of extraction buffer (0.1 M Tris–HCl pH8, 0.05 M EDTA, 1.25% SDS, pre-warmed to 60°C) was added to each well after which the sealing mat was replaced. The block was placed in a custom-made metal clamp to prevent leaking, after which it was incubated for 1–2 h in a water bath at 60°C, with regular mixing by inverting. The block was cooled on ice-water, 150 μL of 6 M ammonium acetate was added and the block was sealed with PCR film. Samples were mixed by inverting 6–8 times and incubated in ice-water for 10 min. Next, the block was centrifuged for 20 min at 4,000 g (4°C) after which 250 μL of the supernatant was transferred to a new masterblock. One hundred and fifty μL of ice-cold 2-propanol was added, the block was sealed with PCR film and mixed by inverting 6–8 times and incubated in ice-water for 10 min. This step was followed by centrifuging for 20 min at 4000 g (4°C) after which the supernatant was poured off and the block dried on tissue paper. 150 μL of 70% ethanol was added, the centrifuge step was repeated and after the supernatant was poured off again the samples were allowed to dry for 5 min at room temperature. Finally, the precipitated DNA was re-dissolved in 400 μL 0.05 M Tris pH9 overnight at 4°C. This method routinely yields 10–30 ng/μL genomic DNA for wheat.

### Endpoint PCR screening

The primer pairs GWDcontrol_for x GWDcontrol_rev and JP_bx17pro5′ x IB_GWD3rev (Table 1) were used for endpoint PCR screening of GWD RNAi transformed plants. The PCR reactions used MyTaq HS (Bioline) and consisted of: 0.5 μL FP (10 μM), 0.5 μL RP (10 μM), 2 μL 5 x buffer, 5.9 μL H_2_O, 0.07 μL MyTaq HS and 1 μL DNA template (ca. 15 ng/μL). The protocol followed the manufacturer’s recommendations with annealing at 58°C. The GWDcontrol primer pair amplifies the endogenous GWD gene, producing a PCR product of ca. 980 bp length (DNA quality control). When present, the primer pair preferentially amplifies a 568 bp product from both the sense and anti-sense sequences, although the endogenous product also remains visible in most cases. The second primer pair is construct-specific, targeting the Bx17 promotor/GWD-sense transition.

**Table 1:** PCR and real-time PCR primers

**Primer name**	**Primer sequence (5′ – 3′)**	**Product size (bp)**
GWDcontrol_for (F)	CGCCTTCTGGCTCAACAGTTC	Endogenous gene; ca. 980
GWDcontrol_rev (R)	TATCACCTTCACCTCCACGAC	Construct: 568
JP_bx17pro5' (F)	AACCATGTCCTGAACCTTCA	606
IB_GWD3rev (R)	ATCTGTAAACCTGTCTTGTG	
qPCR Rint4-9 F	ACATTAGCGAATAGCTGGATGAC	94
qPCR Rint4-9 R	ACATTGATATACTTAGGCACAACCT	
qPCR NOS F	TTGAATCCTGTTGCCGGTCT	127
qPCR NOS R	GCGGGACTCTAATCATAAAAACCC	
qPCR EC ABD F	GGGGAACACTATGGCCTCAA	79
qPCR EC ABD R	TCCAGTTGAATTATCAAGGCCA	
qPCR EC A F	ACCTGACCTTGTAAAACCATTCAT	104
qPCR EC A R	TGACATCCTCCAACATCTCTAAC	
qPCR Rice SPS F	AGAGATCGACGAAAAGCGGA	105
qPCR Rice SPS R	TTTTCGGGATGATCCGAGCC	

F = forward primer, R = reverse primer.

### Real-time PCR primer development

**Figure 1:**
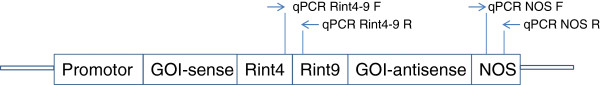
**The wheat RNAi construct showing the two targeted regions for the real-time PCR assay.** To ensure that the real-time PCR primers were specific for the construct but independent of the gene of interest (GOI), the junction between the rice introns (Rint4 and 9) and the NOS terminator were targeted. The shown primer pairs were selected from 3 primer pairs tested for each location, based on specificity and efficiency.

To generate primers that would be useful over the wide range of cereal constructs used in our research group, the boundary between rice intron 4 and 9 (obtained from the Starch Branching Enzyme 1 gene and present in all our cereal RNAi constructs) and the Nopaline Synthase (NOS) terminator (present in all our cereal silencing and over-expression constructs) were selected for primer development (Figure 1). Six primer pairs were trialled for specificity and efficiency (3 per region). Standard measures had to be taken to avoid contamination (e.g. dedicated PCR hood with separate pipettes).

**Figure 2:**
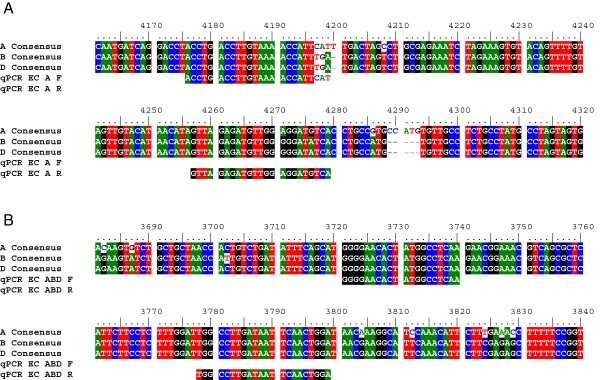
**Alignment of Epsilon Cyclase (EC) fragments of genomes A, B and D from bread wheat.** (**A**) shows priming regions of genome A specific primer pair qPCR EC A and (**B**) shows priming regions of non-genome specific primer pair qPCR EC ABD (F = forward primer, R = reverse primer).

Epsilon Cyclase (EC), a gene involved in the carotenoid biosynthetic pathway [16], was chosen as the wheat reference gene as it is known to be present only once per genome and genome-specific sequences were available. Taking advantage of the hexaploidy of wheat the effectiveness of the primer design and selection procedure was checked. Two sets of three primer pairs were developed and trialled, targeting a conserved domain that was identical between the three genomes (EC ABD), and a specific area for genome A (EC A) (Figure 2). A 1:3 ratio between these two primer pairs would indicate that our primer design and selection process produced primer pairs with matching efficiencies.

All primers were generated using Primer3 software (http://frodo.wi.mit.edu/primer3/) using the following modifications from the default: Product size = 50 – 150 bp; Primer Tm = 59 – 65°C; Product Tm = 80 – 88°C; concentration of divalent cations = 3 mM, concentration of dNTPs = 0.2 mM; Table of thermodynamic parameters = SantaLucia 1998; Salt correction formula = Santalucia 1998.

The trialling of primer pairs consisted of three steps. First, primer pair specificity was determined by using two positive and two negative controls (using the same samples for all primer pairs), selecting for good melt curves (single peak around the expected temperature), whereas no-template controls were used to select primer pairs with no or only low primer dimer formation. If several primer pairs qualified, a pre-selection for efficiency was made by selecting for lowest C_T_ values. The most promising primer pairs were used in sample dilution series using DNA of a single-copy GWD RNAi line (40 ng DNA/μL – 0.1 ng DNA/μL in 3-fold steps). To establish the dynamic range, the highest DNA concentrations of the dilution series are removed when they exhibit higher-than-expected C_T_ values, an indicator of inhibited reactions. Those with the lowest DNA concentrations are removed when they show large variation between replicates, an indicator of the lower detection limit of the real-time PCR technique. The DNA concentration range left after reactions on both ends have been discarded where necessary constitutes the dynamic range and is used in a regression analysis to calculate R^2^ and efficiency values. Lastly, a test sample collection was assayed to determine assay accuracy and precision.

### Real-time PCR copy numbers assay

Reactions were run in a MyIQ real-time PCR machine (BioRAD). A typical reaction consisted of: 10 μl Sensimix SYBR green with Fluorescein, 5 μL primer mix (1.4 μM of both forward and reverse primer), 5 μl DNA template (1 – 6 ng/uL). A standard 2-step protocol for Sensimix was followed: enzyme activation 10 min at 95°C, followed by 40 cycles of 95°C for 15 s, 60°C for 45 s. After each run, a melt-curve was produced in 1°C increments starting at 60°C to check for primer dimer formation. The fluorescent threshold was set at 150 Relative Fluorescent Units (RFU) in all runs. Reactions were run in triplicate. If the standard deviation within a triplicate was above 0.4 of a C_T_, the replicates were screened by eye for obvious outliers, which were removed leaving a duplicate with a standard deviation ≤ 0.4. Samples with a C_T_ standard deviation value above 0.4 with no outliers were removed from further analyses, which occurred rarely. All copy numbers were calculated with the ΔC_T_ method using a reference gene and calibrator sample (essentially the same as the ΔΔC_T_ method [2] but calculated in a different order) on a home-made spreadsheet for the calculations. In short, the “raw” copy numbers were calculated with the formula: raw copy number = 2^(CT(reference)-CT(Gene-Of-Interest))^ × (number of reference gene copies). Next, raw values were divided by the raw value of the calibrator (a known single-copy sample included in all runs) to obtain the (corrected) copy numbers. The advantages of this variant over the standard ΔΔC_T_ method were that (i) in our experimental procedure we found this method more intuitive to calculate, (ii) the raw copy numbers gave additional verification on how well the primer pairs were matched in efficiency (the closer the raw values are to the corrected values, the better the match), and (iii) when single copy samples were identified in a run, dividing the raw values by the mean of the raw values of the single copy samples (including the calibrator) enhanced the precision of copy number determination when compared to just using the raw value of the calibrator.

## Results

### Primer pair selection and sample dilution

**Figure 3:**
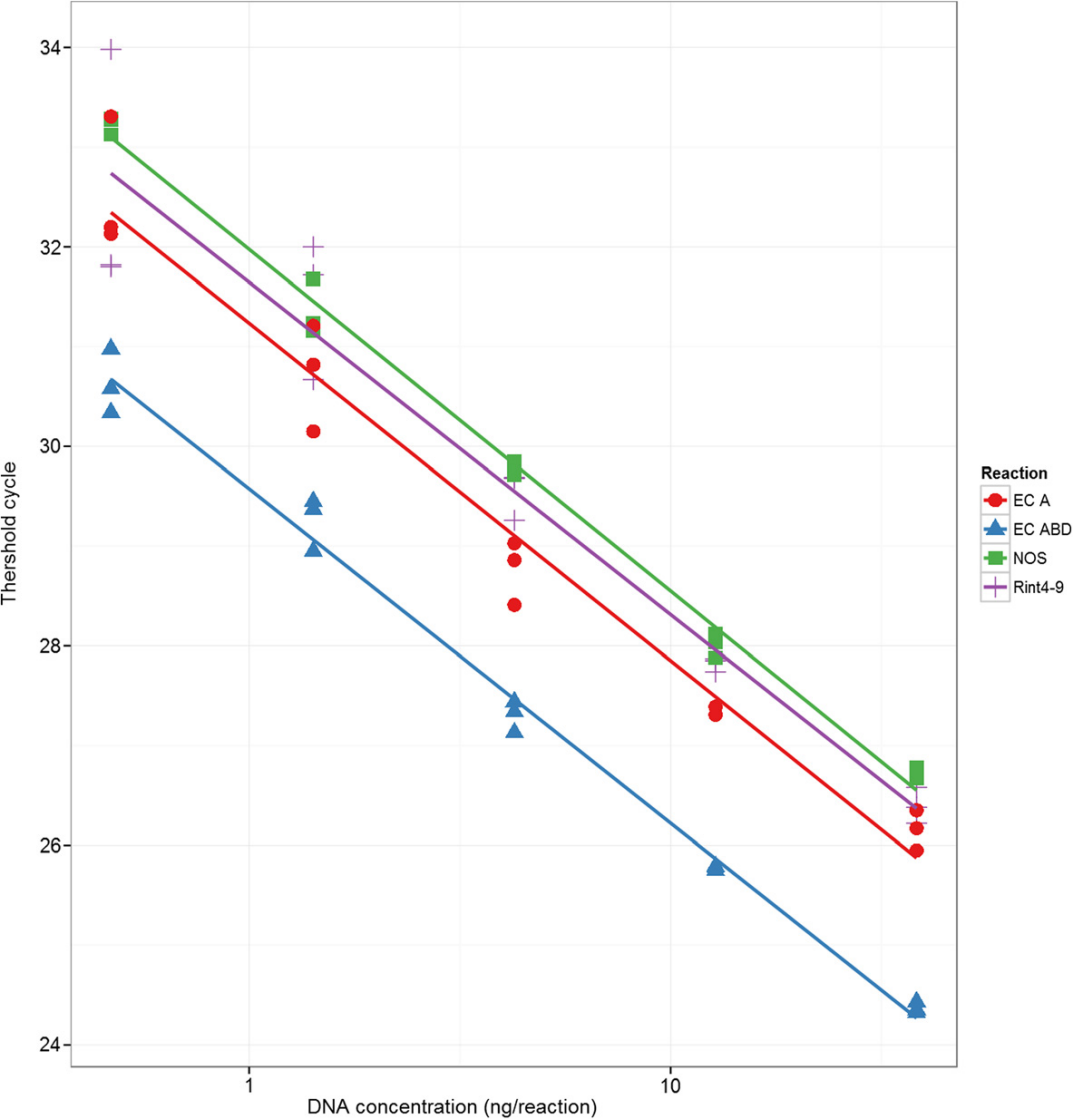
**Sample dilution series on the four primer pairs selected for the copy number assay.** R^2^ > 0.98, efficiency = 100 ± 5% for each dilution series. The highly similar slopes of the fitted lines (produced by regression analyses) indicate that the reaction efficiencies are comparable within the dynamic range.

Four primer pairs were selected after the tests for specificity (clean melt curve and single band on agarose gel) and absence or low level of primer dimers (no or very low signals in the no-template controls): Rint4-9, NOS, EC A, and EC ABD (Table 1). Subsequent sample dilution series and regression analyses showed that their efficiencies were well-matched and close to 100% (Figure 3).

During sample dilutions, it was noted that samples with a DNA concentrations > 13 ng/μL had C_T_ values higher than expected. Similarly, samples with concentrations < 0.3 ng/μL had increased variability between technical replicates. Samples of the dilution series outside the 0.3 – 13 ng/μL range were therefore not included in the regression analyses. Because of this, experimental samples were diluted to stay within a 1–6 ng/ μL range.

A 1 : 3.3 ratio between EC A and EC ABD was obtained. This is close to the expected 1 : 3 ratio and well within the error margin for real-time PCR when comparing two different primer pairs without a reference sample (i.e. a raw copy number). This result further indicates that our primer design and selection process produced well-matched primer pairs.

### Experiment 1

To test if the assay could distinguish between homozygous and hemizygous plants, eight samples were screened using the four selected primer pairs mentioned above. Results are summarized in Table 2. For all the available primer pair combinations, the assay unambiguously identified the four plants with only a single copy, showing a ca. 30% error margin in the copy number estimates (ranging from 0.7 to 1.2). For the homozygous plants, estimates ranged from 1.6 up to 2.6, showing the correct identification with a similar 30% error margin. Correction values (mean of calibrator and single copy samples) were found to vary between runs, indicating some slight variations in efficiencies between different runs, but were always within a 0.6 to 1.8 range.

**Table 2:** Discrimination between hemizygous and homozygous plants

**Sample**	**Rint4-9/EC A**	**NOS/EC A**	**Rint4-9/EC ABD**	**NOS/EC ABD**	**EC A/EC ABD**
**HEM1**	**0.8**	**0.8**	**0.8**	**0.7**	**1.9**
**HEM2**	**0.9**	**0.9**	**1.0**	**0.9**	**2.1**
**HEM3**	**0.9**	**1.0**	**1.0**	**0.9**	**2.1**
**HEM4**	**1.1**	**1.0**	**1.2**	**0.9**	**2.0**
**HOM5**	**2.3**	**2.1**	**2.6**	**2.1**	**2.0**
**HOM6**	**1.9**	**2.0**	**2.0**	**1.9**	**2.0**
**HOM7**	**1.6**	**2.2**	**1.8**	**2.0**	**2.0**
**HOM8**	**1.8**	**1.7**	**2.2**	**1.8**	**2.3**

Headings indicate target regions used in ΔC_T_ analyses, numbers refer to # copies. All comparisons gave similar results (assuming 2 copies for EC A and 6 copies for EC ABD), showing that samples 1–4 were hemizygous and 5–8 were homozygous. The last column shows the number of copies estimated for EC A using EC ABD as the reference.

### Experiment 2

**Figure 4:**
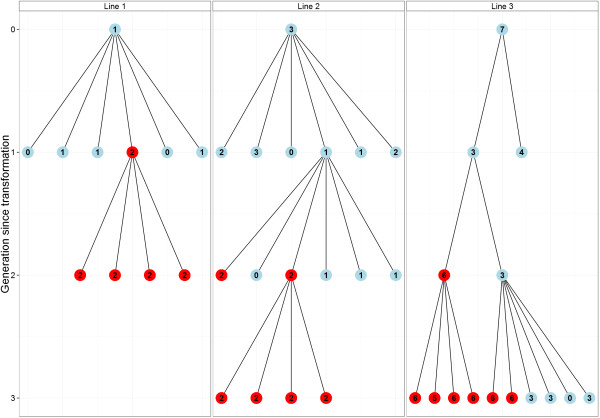
**Development of homozygous wheat lines.** Red indicates identified homozygous plants. Line 1 shows the quickest and preferred way to a homozygous line from a single-copy T_0_ plant, but other paths are possible as shown in lines 2 and 3. Note that for clarity reasons not all plants screened for line 3 are shown.

Three transformed plants were followed over three generations to investigate the effectiveness of the real-time PCR assay in the selection of homozygous transformed lines (Figure 4). The quickest possible route to a homozygous line is demonstrated by line 1, starting with a single copy plant and screening for a homozygous plant in T_1_. When T_0_ is a low copy number (2–3) plant, a single copy plant can often be identified in the T_1_ generation first, after which a homozygous plant can be selected in T_2_ (line 2). However, when starting with a higher copy number T_0_ plant, it may be impossible to select a single copy plant because several copies may be linked, effectively behaving like a single copy. The example of plant line 3 shows that these plants can be identified and be developed into high copy homozygous lines.

The real-time PCR results of experiment 2 were verified in two ways. First, we screened 24 offspring of a hemizygous and a homozygous plant by endpoint PCR. Seven offspring of the single copy plant were negative, whereas all offspring from the homozygous plant were positive, confirming the real-time PCR results. Second, we screened 4 offspring of 3 selected homozygous plants with real-time PCR, confirming that they all possessed the same, even number of copies of the transgene.

### Rice

**Figure 5:**
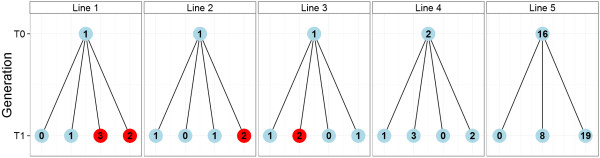
**Development of homozygous rice lines.** Example of a copy numbers screen on rice (T_0_ and T_1_ only) using the Rint4-9 and SPS primer pairs (Table 1). Red indicates identified homozygous plants. Note that multiple insertions prevented the positive identification of a homozygous line in lines 4 and 5 in the T_1_ generation, and that the stability of the identified homozygous lines has not yet been tested in the next generation (T_2_) as is recommended.

We successfully adapted our assay to work on rice. As the rice expression cassette used in the transformation process contained the same standard sequence elements as for wheat, the same construct-specific primer pairs could be used (in this case the Rint4-9 primer pair was chosen). However, a rice reference gene was needed. We selected Sucrose Phosphate Synthase (SPS) because it is a confirmed single-copy gene for which good sequence information is available [17]. Once primer pairs were produced and the best one selected (as described under Methods – Real-time PCR primer development), the assay was as effective for rice as for wheat (Table 1 and Figure 5). Five plants were screened at T_0_ and up to 4 offspring (T_1_) were screened in each case. Homozygous plants carrying a unique insertion were identified in plants 1, 2 and 3, whereas multiple insertions prevented the positive identification of a homozygous line in plants 4 and 5 in the T_1_ generation.

## Discussion

The assay described here provided a powerful tool in the homozygous transgenic line development process. Ideally, the assay is used to select a single copy transgenic T_0_ plant as the assay is most precise in detecting a single copy, and in these lines there is no potential for gene-silencing due to multi-copy insertions. Screening 6 plants of the next generation was generally enough to identify a homozygous plant (expected frequency of 0.25) that could be used as founder of a transgenic homozygous line. Low copy number T_0_ plants can be screened in the next generation for a single copy segregant, which can then be used as the founding plant for a homozygous line carrying a single insertion. In T_0_ plants with multiple copies, back-crossings may be necessary to achieve a single-copy generation (not tested), although this will not work if the plants carry linked copies. A plant with putative linked copies can be made homozygous with a fair degree of certainty, however there remains uncertainty in these analyses about the exact copy numbers which is not present for single insertion homozygous lines (discussed below).

The required high precision was reliably achieved by following a few simple rules: (i) select primers that are (near-)100% efficient (by designing and testing several primer pairs per locus under standardized PCR conditions and selecting the one that is (near-)100% efficient); (ii) use a high-quality commercial qPCR master mix and (self-made) primer pre-mixes to minimize differences between runs by limiting the number of the pipetting steps; (iii) use good quality DNA at the right concentration (within the dynamic range). This approach consistently led to C_T_ values with standard deviations of ≤ 0.4, as is required for copy number and zygosity determinations [13].

An important aspect of the identified ca. 30% error margin in these analyses is that there is very little uncertainty about the exact copy number of a single copy plant, but this uncertainty increases incrementally with increasing copy numbers. For instance, a result of 6 copies should be interpreted as having between 4 and 8 copies. This error margin may be reduced by running more technical replicates (not tested); however, for our analyses we deemed this not necessary as the main aim was to select plants with a unique insertion as efficiently as possible. To reflect this error margin, copy number results should be reported as integers. Additionally, to minimize the possibility of false positives, several offspring of an identified homozygous plant should be screened to verify copy number stability.

Bubner and Baldwin [2] showed that most previous studies have used Taqman probes for copy number analyses (one exception being [12]), suggesting that this is because these probes are superior since they are not affected by primer dimer formation. However, we considered that SYBR green has an important advantage over Taqman probes for copy number analyses and the focus on Taqman probes may be one of the reasons why previous studies have struggled to get to the required accuracy for reliable zygosity screening. In our experience, the use of SYBR green facilitates the selection of primer pairs that can amplify at near-100% efficiency. The much lower cost per primer pair makes it more feasible to order several primer pairs per target. Also, the chance of finding a suitable DNA sequence for your primers is higher with SYBR green (especially important when working within a small region) because the product can be anywhere between 60 and 200 bp and does not need to be suitable for a probe. Rather than optimising the PCR reaction to the primers, the PCR conditions are standardised and only primer pairs are selected that amplify with near-100% efficiency under the standard conditions and do not produce interfering primer dimers.

The one benefit of Taqman probes over SYBR in copy number determinations is the ability they provide for multiplexing, which is a powerful advantage for high-throughput screening. However, our experience is that the process of selecting homozygous lines is not a high-throughput procedure per se, but rather benefits from the flexibility to screen for many different constructs and easy adaptation procedures to new targets, as is the case for our assay. Any wheat construct containing one of the two standard sequence elements can be screened using the primers provided, and the example of rice indicates that the assay can easily be adapted to other plants.

Because bread wheat is hexaploid, we were able to make genome non-specific and genome specific primer pairs for the reference gene. Either pair could be used as a reference, but it is important to know how many copies the reference primer pair amplifies, because this will affect the raw transgene copy number estimate. Other organisms such as rice and barley do not have this complication and primer design is therefore somewhat easier.

Fitzgerald *et al.*[18] have used genome-specific real-time PCR in wheat to detect Null-plants in large sample collections (plants with the gene of interest missing in one of the three genomes). We propose that our method can assist in the selection of double (and triple) null lines through crosses of these single null plants. With our method it should be possible to select the plants that are null for 1 genome and hemizygous for the other, which could be of great benefit especially if double nulls are elusive (e.g. due to negative selection pressure). A similar advantage can be gained when crossing multiple constructs into a single plant line (using construct-specific primer pairs). Finally, our assay could be useful in investigating endogenous gene copy number variation for low copy number genes.

## Conclusions

Here we present a real-time PCR based method to fast-track the development of transgenic homozygous lines in commercially important plant species with relatively long generation times, such as wheat and rice. Standardized procedures for DNA isolation, primer design, primer selection and real-time PCR resulted in a method that is able to reliably distinguish single copy from low to high copy number plants, which can be done as early as the T_0_ generation. Zygosity determinations can be performed on their descendants to select homozygous lines carrying a single insertion, avoiding potential issues with transgene silencing and complex deregulation procedures for commercial use associated with plants with multiple insertions. We specifically designed the method to be relatively quick, affordable and versatile so it can be used with many different cereal constructs and may easily be adapted to different vectors and plant species. SYBR green is considered to have several advantages over Taqman probes for copy number analyses, including benefits in primer design, flexibility and cost-effectiveness. The method greatly assisted in the development of homozygous lines and promises a significant saving in time and collection management efforts.

## Competing interests

The authors declare that they have no competing interests.

## Authors’ contributions

JM conceived the study, participated in the design, carried out the molecular studies and drafted the manuscript. JR and CH participated in the design of the study and helped to draft the manuscript. All authors read and approved the final manuscript.

## References

[B1] Lemaux PG Genetically engineered plants and foods: A scientist’s analysis of the issues (Part I) Annu Rev Plant Biol 2008 59 771 812 18284373 10.1146/annurev.arplant.58.032806.103840

[B2] Bubner B Baldwin IT Use of real-time PCR for determining copy number and zygosity in transgenic plants Plant Cell Rep 2004 23 5 263 271 15368076 10.1007/s00299-004-0859-y

[B3] Butaye KMJ Goderis IJWM Wouters PFJ Pues JMTG Delaure SL Broekaert WF Depicker A Cammue BPA De Bolle MFC Stable high-level transgene expression in Arabidopsis thaliana using gene silencing mutants and matrix attachment regions Plant J 2004 39 3 440 449 15255872 10.1111/j.1365-313X.2004.02144.x

[B4] Sood P Bhattacharya A Sood A Problems and possibilities of monocot transformation Biol Plantarum 2011 55 1 1 15

[B5] Tang W Newton RJ Weidner DA Genetic transformation and gene silencing mediated by multiple copies of a transgene in eastern white pine J Exp Bot 2007 58 3 545 554 17158108 10.1093/jxb/erl228

[B6] Bhat SR Srinivasan S Molecular and genetic analyses of transgenic plants: Considerations and approaches Plant Sci 2002 163 4 673 681

[B7] Gadaleta A Giancaspro A Cardone MF Blanco A Real-time PCR for the detection of precise transgene copy number in durum wheat Cell Mol Biol Lett 2011 16 4 652 668 21922222 10.2478/s11658-011-0029-5 PMC6275630

[B8] Li ZW Hansen JL Liu Y Zemetra RS Berger PH Using real-time PCR to determine transgene copy number in wheat Plant Mol Biol Rep 2004 22 2 179 188

[B9] Shou HX Frame BR Whitham SA Wang K Assessment of transgenic maize events produced by particle bombardment or Agrobacterium-mediated transformation Mol Breeding 2004 13 2 201 208

[B10] Yang LT Ding JY Zhang CM Jia JW Weng HB Liu WX Zhang DB Estimating the copy number of transgenes in transformed rice by real-time quantitative PCR Plant Cell Rep 2005 23 10–11 759 763 15459795 10.1007/s00299-004-0881-0

[B11] German MA Kandel-Kfir M Swarzberg D Matsevitz T Granot D A rapid method for the analysis of zygosity in transgenic plants Plant Sci 2003 164 2 183 187

[B12] Casu RE Selivanova A Perroux JM High-throughput assessment of transgene copy number in sugarcane using real-time quantitative PCR Plant Cell Rep 2012 31 1 167 177 21953330 10.1007/s00299-011-1150-7

[B13] Bubner B Gase K Baldwin IT Two-fold differences are the detection limit for determining transgene copy numbers in plants by real-time PCR BMC Biotechnol 2004 4 14 15251044 10.1186/1472-6750-4-14 PMC493272

[B14] Ral JP Bowerman AF Li Z Sirault X Furbank R Pritchard JR Bloemsma M Cavanagh CR Howitt CA Morell MK Down-regulation of Glucan, Water-Dikinase activity in wheat endosperm increases vegetative biomass and yield Plant Biotechnol J 2012 10 7 871 882 22672098 10.1111/j.1467-7652.2012.00711.x

[B15] Ellis MH Rebetzke GJ Azanza F Richards RA Spielmeyer W Molecular mapping of gibberellin-responsive dwarfing genes in bread wheat Theor Appl Genet 2005 111 3 423 430 15968526 10.1007/s00122-005-2008-6

[B16] Cunningham FX Gantt E Genes and enzymes of carotenoid biosynthesis in plants Annu Rev Plant Phys 1998 49 557 583 10.1146/annurev.arplant.49.1.557 15012246

[B17] Jiang LX Yang LT Zhang HB Guo JC Mazzara M Van den Eede G Zhang DB International collaborative study of the endogenous reference gene, sucrose phosphate synthase (SPS), used for qualitative and quantitative analysis of genetically modified rice J Agr Food Chem 2009 57 9 3525 3532 19326953 10.1021/jf803166p

[B18] Fitzgerald TL Kazan K Li ZY Morell MK Manners JM A high-throughput method for the detection of homoeologous gene deletions in hexaploid wheat BMC Plant Biol 2010 10 264 21114819 10.1186/1471-2229-10-264 PMC3017838

